# Ghrelin, a gastrointestinal hormone, regulates energy balance and lipid metabolism

**DOI:** 10.1042/BSR20181061

**Published:** 2018-09-25

**Authors:** You Lv, Tingting Liang, Guixia Wang, Zhuo Li

**Affiliations:** 1 Department of Endocrinology and Metabolism, The First Hospital of Jilin University, Changchun, Jilin, China; 2 Department of Oncology, The First Hospital of Jilin University, Changchun, Jilin, China

**Keywords:** energy homeostasis, Ghrelin, GHSR, hypothalamus, lipid metabolism

## Abstract

Ghrelin, an acylated peptide hormone of 28 amino acids, is an endogenous ligand of the released growth hormone secretagogue receptor (GHSR). Ghrelin has been isolated from human and rat stomach and is also detected in the hypothalamic arcuate nucleus. Ghrelin receptor is primarily located in the neuropeptide Y and agouti-related protein neurons. Many previous studies have shown that ghrelin and GHSR are involved in the regulation of energy homeostasis, and its administration can increase food intake and body weight gain. AMP-activated protein kinase is activated by ghrelin in the hypothalamus, which contributes to lower intracellular long-chain fatty acid level. Ghrelin appears to modulate the response to food cues via a neural network involved in the regulation of feeding and in the appetitive response to food cues. It also increases the response of brain areas involved in visual processing, attention, and memory to food pictures. Ghrelin is also an important factor linking the central nervous system with peripheral tissues that regulate lipid metabolism. It promotes adiposity by the activation of hypothalamic orexigenic neurons and stimulates the expression of fat storage-related proteins in adipocytes. Meanwhile, ghrelin exerts direct peripheral effects on lipid metabolism, including increase in white adipose tissue mass, stimulation of lipogenesis in the liver, and taste sensitivity modulation.

## Introduction

Ghrelin is an acylated peptide hormone that was first described in 1999 as the endogenous ligand of released growth hormone secretagogue receptor (GHSR). It is a 28-amino acid peptide, in which the serine 3 residue is *n*-octanoylated. Ghrelin has been isolated from the secretory granules of X/A-like cells in the submucosal layer of the stomach; it is also detected in the hypothalamic arcuate nucleus and regulates energy balance [1]. The X/A-like cells contain round, compact, electron-dense granules filled with ghrelin [2]. Ghrelin is also synthesized in the placenta, kidney, heart, and thyroid [3]. Ghrelin was named after the Proto-Indo-European root ‘g^h^re’ meaning ‘to grow’ as ghrelin exhibits potent growth hormone (GH)-releasing activity [1].

Ghrelin exists in two forms: acylated ghrelin (AG) and unacylated ghrelin. *O*-acyltransferase (GOAT) transfers an acyl group from the fatty acids to the serine-3 residue of ghrelin and, thus, stimulates GH secretion and food intake [4,5]. GOAT activates ghrelin depending on an esterification reaction, and blockade of GOAT activity induces less food intake and weight gain [4,6]. Therefore, GOAT may offer a therapeutic target for obesity.

GH is synthesized by somatotroph cells of the anterior pituitary. GH induces protein synthesis, nitrogen retention, and impairs glucose tolerance by antagonizing insulin action. Moreover, GH plays a very important role in lipolysis and maintains water–mineral metabolism balance. Linear bone growth is mediated by a complex network of hormones and growth factors, including insulin-like growth factor-I. GH secretion is controlled by GH-releasing hormone (GHRH). GHSR is distinct from the GHRH receptor, and it has been demonstrated that GHS acts through GHSR, thus, constituting an entirely novel mechanism of regulating GH secretion. Ghrelin is the natural endogenous ligand for GHSR [1,7]. In humans, the GHSR1 gene encodes full-length GHSR1a and the truncated isoform GHSR1b [8,9]. AG is necessary for binding to GHSR-1a [4,10]. A previous study has shown that ghrelin receptor is primarily located in the neuropeptide Y (NPY) and agouti-related protein (AGRP) neurons of the hypothalamus–pituitary unit [11]. However, recent results have demonstrated that 17.6% of the somatostatin (SST) neurons in the tuberal nucleus (TN) were GHSR-positive, with the most caudal TN containing fewer GHSR-positive SST neurons [12]. Loss-of-function experiments using ghrelin knockout (KO) mice [13] or ghrelin receptor KO mice [14] have shown that lack of ghrelin function could protect against early-onset obesity.

Ghrelin is usually described as an orexigenic hormone whose concentration increases during calorie restriction [15]. New updated studies have indicated that ghrelin appears to modulate the response to food cues via a neural network involved in the regulation of feeding, most importantly in the appetitive response to food cues. This appetitive response has several components: attention, anticipation of pleasure, motivation to eat, consumption, and memory for associated cues. This response to food pictures are located in the amygdala, orbitofrontal cortex (OFC), insula, visual areas, and striatum [15], and these regions encode the salience, hedonic, and incentive value of visual cues. This effect may account for the effect of ghrelin to stimulate food intake [16]. Moreover, other actions of ghrelin include stimulation of gastric emptying and motility, modulation of sleep, increased taste sensation, regulating glucose and adipose metabolism, protection against muscle atrophy, and improved cardiovascular function [8,15].

## Function of ghrelin and its receptor

Ghrelin, a hormone produced in the stomach, exhibits orexigenic properties and has recently attracted enormous interest as a potential anti-obesity therapeutic target [17]. Chronic ghrelin administration promotes weight gain and adiposity [18]. Moreover, obese patients diagnosed with Prader–Willi syndrome are characterized by increased circulating levels of AG with a relative deficit of unacylated ghrelin (also known as desacyl ghrelin); unacylated ghrelin analog improves postprandial glucose levels and decreases waist circumference and fat mass in humans [19,20]. These findings indicate that ghrelin is a powerful endogenous orexigenic peptide.

Ghrelin levels can be influenced by multiple factors, including diet composition, exercise, environment, and lifestyle. Jakubowicz et al. [21] found that compared with a high-carbohydrate diet, ghrelin was significantly suppressed by whey-protein diet in patients with Type 2 diabetes mellitus (T2DM). However, Giezenaar et al. [22] found that protein drinks cause load-dependent slowing of gastric emptying and increase plasma ghrelin concentrations in older individuals. A recent study revealed that reducing dietary energy density promotes weight loss by inhibiting ghrelin in obese women [23]. Ghrelin could also be suppressed by a hypocaloric diet, polyunsaturated fat-rich diet, and fructose supplementation [24–26]. Low meal frequency increases fasting plasma ghrelin concentration more than that by the same caloric restriction split into six meals in patients with T2DM [27]. AG can be decreased by intense aerobic exercise and very low-intensity intermittent exercise [28,29]. Ghrelin level increases after sleep restriction compared with that after normal sleep and is associated with more consumption of calories; thus, elevated ghrelin may be a mechanism by which sleep loss leads to increased food intake and obesity [30]. In recent years, short-chain fatty acids (SCFAs) have been found to influence appetite hormone secretion in animals and humans. Acute increased colonic SCFAs do not affect glucagon-like peptide-1 (GLP-1) or peptide YY (PYY) responses but reduce ghrelin levels [31]. Exposure to heat induces a decrease in plasma ghrelin level [32]. However, Kiessl et al. [33] found that stress does not affect postprandial ghrelin secretion in women. Therefore, through the alteration of ghrelin, metabolic dysfunction may be regulated.

Most studies have reported higher postprandial repressions of ghrelin secretion following bariatric surgeries that restrict the size of the stomach, including Roux-en-Y gastric bypass (RYGB), sleeve gastrectomy (SG), and biliopancreatic diversion with duodenal switch (BPD/DS) [34–36]. Cummings et al*.* [19] investigated plasma ghrelin levels after weight loss induced by diet or gastric bypass surgery and found that gastric bypass decreases ghrelin levels, possibly contributing to the weight-reducing effect of the procedure. Tamboli et al. [10] studied the central and peripheral responses to ghrelin in obesity and during the early period after RYGB in humans and found that ghrelin-stimulated GH secretion is attenuated in obesity, and this effect improves 2 weeks after RYGB, but peripheral insulin sensitivity is not altered. This suggested that the effects of ghrelin in obesity are primarily characterized by central regulation but not peripheral. A previous study also indicated that fasting ghrelin levels are higher in BPD/DS and SG rats than those in sham animals [37]. Studies on BPD/DS have also produced inconsistent results regarding ghrelin secretion [36,38], and there have been some controversial results recently. Kalinowski et al. [39] compared the effects of SG and RYGB on ghrelin, leptin, and glucose homeostasis in a randomized controlled trial and found that ghrelin levels decrease after SG but increase after RYGB; however, metabolic improvements are not affected. Casajoana et al. [40] also found that ghrelin levels are higher in the RYGB group than those in the SG and greater curvature plication groups at 1 year. The mechanism may be due to a compensatory increase in ghrelin secretion as an adaptive response that constrains weight loss. In summary, the present studies indicated that bariatric surgeries lead to metabolic improvement, and one of the mechanisms may be the alteration of ghrelin levels. Synthetic human ghrelin when administered to patients with severe body weight reduction after gastrectomy presented a gain in body weight and appetite improvement [41].

Ghrelin enhances food intake in a dose-dependent manner in rats. In humans, intravenous administration of ghrelin at physiological doses induces hunger and short-term enhancement in food intake [42]. Higher unacylated ghrelin levels could predict higher weight loss in humans [43]. In healthy individuals, continuous infusion of AG decreases blood pressure, mean arterial blood pressure, heart rate, and body surface temperature compared with that by desacyl ghrelin infusion, which may be related with modulation of the autonomic nervous system [44]. Intraperitoneal ghrelin injection to mice enhances glucose-stimulated GLP-1 release and improves glucose tolerance [45]. Ghrelin concentration increases to almost 2-fold before a meal and then rapidly decreases after the meal. However, a recent study also revealed that a fleeting rise in AG concentration is observed after a meal and then decreases in normal-weight and obese men [46]; however, the mechanism has not been completely elucidated. One reason may be that perhaps ghrelin level is associated not only with consuming food but also with viewing or smelling food, ingestion, and food variety [46]. The vagus nerve is an important mediator in the ghrelin action pathway [18,42]. Previous studies have also established that ghrelin is involved in the regulation of energy homeostasis [18].

Ghrelin activates hypothalamic AMPK to perform its orexigenic action. Central injection of Ex527, a sirtuin 1 inhibitor, or genetic depletion of p53 leads to a failure by ghrelin to phosphorylate hypothalamic AMPK and reduces ghrelin-induced food intake in rats [47]. The same study also revealed that chronic peripheral ghrelin administration improves ghrelin-induced gain in body weight and fat mass in P53-null mice and blocks the lipogenic effect in white adipose tissue (WAT) and the liver [48]. These data demonstrated that the hypothalamic sirtuin 1/p53 pathway is crucial for the orexigenic effect of ghrelin. Central cannabinoid receptor type 1 participates in the signaling pathway that mediates the effects of ghrelin on AMPK in peripheral tissues [49]. The ghrelin–AMPK signaling pathway also exhibits a protective effect in substantia nigra dopamine neurons during calorie restriction in Parkinson’s disease [50].

GHSR is a G-protein coupled receptor and exhibits strong homology across species. GHSR–ghrelin binding activates the phospholipase C signaling pathway through Gq, leading to protein kinase C activation, followed by release of Ca^2+^ from intercellular stores and diacylglycerol production [51]. The ghrelin receptor also co-couples with Gs protein, activating the cAMP/cAMP responsive element-binding protein pathway [52]. Moreover, the ghrelin receptor can also interact with GPR83, and GPR83/GHSR1a dimerization affects the ability of ghrelin to activate its endogenous receptor [53].

Jeffrey et al. [14] found that ghrelin administration failed to acutely stimulate food intake or activate arcuate nucleus neurons in a GHSR-null mouse model. Similar effects on body weight and adiposity were also observed in female mice, but not in male mice. These results suggest that ghrelin and GHSR are important regulators of food intake and body weight control. Moreover, their data suggest that ghrelin signaling is required for the development of the full phenotype of diet-induced obesity. Mice lacking ghrelin receptors resist the development of diet-induced obesity. Ghrelin inhibits glucose-stimulated insulin secretion (GSIS). A recent research indicated that the anorexic hormone obestatin increases GSIS, and this effect is also mediated by GHSR [54]. The effects of GHSR are associated with peripheral energy balance and central regulation. By increasing AG levels via injection and/or calorie restriction (CR) in GHSR-eGFP mice, the expression of neurogenic transcription factor increases in the dentate gyrus (DG), and 2 weeks of CR induce a significant increase in new neurons in the DG of wild-type but not GHSR^−/−^ mice [55]. It has been demonstrated that GHSR is essential for hippocampal plasticity. Ghrelin receptor also heterodimerizes with dopamine receptor-1 (DRD1) in hippocampal neurons, and GHSR inactivation completely attenuates DRD1-regulated hippocampal behavior and memory [56]. The ghrelin receptor agonist relamorelin accelerates the frequency of distal antral motility contractions without significant effects on the amplitude of contractions in healthy volunteers [57]. Relamorelin also significantly reduces symptoms of diabetic gastroparesis and accelerates gastric emptying [58]. A 3-month randomized, crossover, double-blind clinical study on an oral ghrelin receptor agonist, GRA, showed that GRA increases IGF-1 level in hemodialysis patients with protein–energy wasting [59]. These results suggest that the ghrelin receptor agonist may be used for patients with chronic kidney diseases and symptomatic gastroparesis in the future, but more clinical studies are still needed. In pharmacokinetics and pharmacodynamics of the clinical trial, ghrelin receptor inverse agonists acutely block the ghrelin receptor in healthy volunteers and dose-dependently increase heart rate, delay gastric emptying, and induce somnolence [60]. Unacylated ghrelin analog is well tolerated in humans and exhibits metabolic benefits in overweight/obese and T2DM subjects [61].

## Role of ghrelin in the regulation of energy expenditure

Serum ghrelin may play a role in the regulation of energy expenditure through the induction of metabolic changes that would lead to an efficient metabolic state, resulting in increased body weight and fat mass (Table 1).

**Table 1:** Effects of ghrelin on energy regulation

Research subjects	Treatment	Mode of administration	Energy regulation and metabolism
Wistar rats [7]	Rat ghrelin	ICV ghrelin injection: 3, 10, 50, 200, 500, and 1 nmol	Ghrelin increases food intake and body weight gain.
			The plasma concentrations of glucose, insulin, triglycerides, and total cholesterol in the ghrelin-infused group did not differ from those of the control group.
GHSR-null mice [14]	Rat/mouse ghrelin	ICV ghrelin injection	Ghrelin stimulates food intake in wild-type mice but not in GHSR-null mice.
			The arcuate nucleus neurons normally activated by ghrelin are not activated in GHSR-null mice.
			When fed with an HFD, GHSR-null mice consume less food and store less consumed calories.
Mice and rats [18]	Rat ghrelin	Subcutaneous ghrelin injection: 2.4 µmol/kg/day	Peripheral ghrelin injection induces an increase in weight gain by reducing fat utilization in mice and rats.
		ICV ghrelin injection: 1.2 nmol/kg/day or 12 nmol/kg/day	ICV ghrelin injection induces a dose-dependent increase in body weight.
Sprague–Dawley rats [62]	Rat ghrelin	ICV ghrelin injection: 1 µg/rat	Chronic central administration of rat ghrelin increases food intake and body weight and does not affect plasma insulin, glucose, leptin, or GH concentrations.
C57Bl6 mice [63]	Ghrelin	Intraperitoneal injection of ghrelin: 1 mg/kg	Exogenous ICV ghrelin does not induce food intake in DIO mice.
		ICV injection of ghrelin: 1 µg/µl	DIO decreases expression of NPY and AGRP mRNA, and central ghrelin is unable to promote expression of these genes.
			DIO causes central ghrelin resistance by reducing NPY/AGRP responsiveness to plasma ghrelin and suppresses the neuroendocrine ghrelin axis to limit further food intake.
Ghrelin knockout mice [65]	Ghrelin	Intraperitoneal injection of ghrelin: 1, 6, 15, 30, 50, or 75 nmol/kg	Food intake in ghrelin^−/−^ mice is not different from that in ghrelin^+/+^ mice during normal light-cycle conditions.
		Intraperitoneal injection of des-octanoyl ghrelin: 15 or 30 nmol/kg	Interruption in the normal light/dark cycle triggers additional food intake in old ghrelin^+/+^ mice but not in ghrelin^−/−^ mice.
			Exogenous ghrelin increases food intake in both genotypes with a bell-shaped dose–response curve that shifts to the left in ghrelin^−/−^ mice.
*Syn1-Cre;Ghsr^f/f^*mice [66]	GHSR gene was deleted in all neurons using Synapsin 1-Cre driver.	Ghrelin-induced spontaneous food intake: after 3 h of fasting (7:00 to 10:00 AM), mice were i.p. injected with physiologic saline, and then food intake was measured. After 30 min, the same mice were i.p. injected with ghrelin at 0.5 mg/kg of body weight.	Neuronal GHSR deletion abolishes ghrelin-induced spontaneous food intake, prevents DIO, and improves insulin sensitivity but has no effect on total energy intake.
Adults [67]	During energy balance (EB), energy intake is equivalent to the energy expenditure to maintain EB. During energy deprivation (ED), energy intake is <10% of that during EB.	EB: 85% carbohydrate, 13% fat, and 2% protein ED: 66% carbohydrate, 20% fat, and 12% protein	Short-term, severe ED suppresses AG concentrations and increases postprandial insulin, GLP-1, and PP concentrations.
Obese and lean normoglycemic Chinese men [68]	High-protein (HP) meal	An isocaloric (approximately 600 kcal) isovolumic (approximately 400 ml) liquid meal was given to the subjects to be ingested within 5 min.	Postprandial GLP-1 response after HF or HP meal was higher than that of HC meal in both lean and obese subjects. In obese subjects, HF meal induced higher response in postprandial PYY compared with that by HC meal.
	High-carbohydrate (HC) meal		HP and HF meals exhibited higher suppression of ghrelin compared with that by HC meal in obese subjects than that in lean subjects.
	High-fat (HF) meal		
Adults [69]	A 750-kcal drink with the same protein content while consuming either 20 energy-percent (E%) or 55 E% from carbohydrates and the remaining energy from fat.	Participants were randomized to consume the drinks as one large beverage or as five 150-kcal portions every 30 min.	Energy expenditure (EE) was higher after the high-carbohydrate drinks and also after ingesting one drink compared with that after five drinks.
			Serum ghrelin levels were suppressed 1.5 h after ingestion of the first beverage, but the area under the curves (AUCs) did not differ.
			Serum ghrelin levels were suppressed 2.5 h after low-carbohydrate drinks and also exhibited more sustained appetite suppression than that by high-carbohydrate beverages.
Overweight/obese adults [70]	A breakfast meal containing walnuts	Participants were instructed to consume the test meal within 20 min	GLP-1 was lower after consuming the walnut-containing meal at 60 min.
	A meal without walnuts		Postprandial PYY, ghrelin, and cholecystokinin levels did not differ between the meals at any of the time points.

Ghrelin is the only anabolic gastrointestinal hormone that has been detected so far. Its action has been studied in regulating body weight. Under normal circumstances, a balance in energy intake and energy expenditure results in body weight maintenance. Weight loss would occur if there is a relative increase in energy expenditure and/or a relative decrease in food intake. On the contrary, weight gain would be expected if there is a relative increase in food intake and/or a relative decrease in energy expenditure. Very shortly after its discovery, it was reported that intracerebroventricular (ICV) injection of ghrelin strongly stimulates feeding in rats. As predicted for an orexigenic agent that also decreases energy expenditure, administered ghrelin produces a positive energy balance and induces increased body weight gain [7,18].

So far, investigators have found that the effects of ghrelin on body weight probably include many actions in addition to its direct effect on food intake and decreased energy expenditure. Chronic ICV injection of ghrelin increases cumulative food intake and decreases energy expenditure, resulting in body weight gain [62]. These effects of energy partitioning indirectly influence other factors in the energy balance equation. Different results have been reported in high-fat diet (HFD) animals; Briggs et al*.* [63] found that in HFD animals, both total and active ghrelin decrease and diet-induced obesity (DIO) suppresses GHSR mRNA expression in the hypothalamus. Moreover, exogenous ICV ghrelin does not induce food intake in DIO mice. NPY and AGRP mRNA expression also decrease in DIO mice. Therefore, they suggested that DIO impairs ghrelin-induced release of NPY and AGRP in the hypothalamus to cause ghrelin resistance. Recent reports have shown that central ghrelin administration increases the respiratory quotient (RQ), thus, indicating decreased use of lipids for the generation of energy. Furthermore, ghrelin increases the expression of fat storage enzymes in WAT and decreases the expression of thermogenesis-related uncoupling of proteins in brown adipose tissue [64]. Importantly, the latter effects are shown to occur after ICV administration of ghrelin and are independent of ghrelin-induced hyperphagia.

For further clarification of the effects of endogenous ghrelin in the regulation of energy homeostasis and gastric emptying, ghrelin knockout mice (ghrelin ^(−/−)^) were generated [65]. Although body weight gain and 24-h food intake remained unaffected, during the dark period, young ghrelin ^(−/−)^ mice exhibited a lower RQ, whereas their heat production was higher than that of the wild-type littermates, inferring a role of ghrelin in the regulation of energy expenditure. These data show that when fed with an HFD, both female and male GHSR-null mice consume less food, store less consumed energy, preferentially utilize fat as an energy substrate, and accumulate less body weight and adiposity than control mice. These results suggest that ghrelin signaling is required for the control of energy expenditure [14]. Lee et al. [66] generated a mouse line where the GHSR gene was deleted in all neurons. The results showed that neuronal GHSR gene deletion destroys ghrelin-induced food intake and DIO markedly improves insulin sensitivity.

A randomized, controlled, crossover clinical trial revealed that short-term, severe energy deprivation (ED) inhibits AG concentrations and increases postprandial anorexigenic hormone concentrations (including GLP-1, pancreatic polypeptide, and insulin levels), suggesting an adaptive counterregulatory response to ED in non-obese adults [67]. Rizi et al. [68] reported that a high-protein or high-fat meal suppresses ghrelin and induces more favorable postprandial satiety than that by a high-carbohydrate meal in insulin-resistant individuals. Low-carbohydrate drinks could decrease serum ghrelin levels after 2.5 h than those by high-carbohydrate drinks, and meal frequency also affects the sense of satiety [69]. However, Rock et al. [70] showed that a nut-containing meal does not contribute to increased satiety due to gastrointestinal hormone response (including GLP-1, PYY, pancreatic polypeptide, cholecystokinin, and ghrelin).

Ghrelin plays an important role in the regulation of appetite and energy expenditure; therefore, inhibitors of the ghrelin system are an attractive target for anti-obesity therapies. Patterson et al*.* [71] reviewed the application of ghrelin system antagonist in obesity treatment. The approaches included antagonizing the ghrelin receptor, neutralizing circulating ghrelin, and inhibiting ghrelin *O*-acyltransferase. Some studies have shown that antagonizing the ghrelin system can reduce body weight for a short term [72], but more studies are required to determine its effect in the long term. Most of the approaches are premature and particularly in the clinical trial stage.

## Hypothalamic regulator of food intake and energy balance

The hypothalamus is considered as the key region in the central nervous system (CNS) and is involved in the feedback control of appetite and food intake, although other brain regions have also been implicated. There are two systems that regulate the quantity of food intake: (1) short-term regulation, which is primarily concerned with preventing overeating during each meal; (2) long-term regulation, which is primarily related with the maintenance of normal quantities of energy stores in the form of fat in the body [73]. In short-term regulation of energy intake, the structures in the brain control the intake of a single meal regarding its volume, energy content, and duration. In the long-term coordination of dietary intake and energy expenditure, the CNS receives numerous impulses and peripheral signals, particularly from the gastrointestinal tract and fat tissue in response to constantly altered balance.

The major site of proopiomelanocortin (POMC) expression in the CNS originates in neurons of the arcuate nucleus; most POMC-positive cells also express the anorectic peptide cocaine amphetamine-related transcript (CART). POMC- and CART-positive (POMC/CART) cell bodies are found throughout the rostrocaudal extent of the arcuate nucleus and periarcuate area of the hypothalamus [74]. Arcuate POMC cells send a dense bundle of fibers ventrally to other brain regions, such as the thalamus and mesolimbic area. As arcuate NPY/AGRP-expressing neurons express the potent melanocortin-3 and -4 receptor antagonist, AGRP, they are also a critical component of the central melanocortin system because they are the target of various peripheral hormonal signals, such as ghrelin, leptin, and insulin [75]. AGRP-immunoreactive fibers primarily appear in a subset of the same hypothalamic and septal brain regions containing dense POMC innervation, with the densest fibers found innervating the paraventricular, dorsomedial hypothalamus, posterior hypothalamus, and septal regions around the anterior commissure [76]. The interaction of both POMC/CART and NPY/AGRP systems seems to be the primary driving force in the regulation of energy homeostasis [74].

It has been demonstrated that ghrelin is involved in hypothalamic regulation of energy homeostasis. The action of ghrelin is critical for the normal development of hypothalamic neural circuits during early life [77]. ICV injection of ghrelin markedly stimulates food intake and increases body weight in rats and increases feeding in GH-deficient rats. After ICV ghrelin administration, Fos protein, which is a marker of neuronal activation, has been found in the regions of feeding regulation, including NPY and AGRP neurons. Ghrelin augments NPY gene expression and blocks leptin-induced feeding reduction. Taken together, the authors concluded that ghrelin is a physiological mediator of feeding and probably functions in growth regulation by stimulating feeding and GH release [7].

Lopez et al. [78] used pharmacological and genetic approaches to demonstrate that the physiological orexigenic response to ghrelin involves specific inhibition of fatty acid biosynthesis induced by AMPK resulting in decreased hypothalamic levels of malonyl-CoA and increased carnitine palmitoyltransferase 1 activity. Their results indicated that the metabolism of hypothalamic fatty acid in response to ghrelin is a physiological mechanism that controls feeding regulation. Inhibition of the action of ghrelin in neonatal mice results in enhanced arcuate nucleus neural projections and lifelong metabolic dysfunction, including higher body weight, elevated visceral fat accumulation, increased blood glucose levels and food intake, and decreased leptin sensitivity [77]. The mechanism of ghrelin resistance may include defective ghrelin transport into the hypothalamus [79]. These studies demonstrated that ghrelin plays a crucial role in hypothalamic circuits and metabolic regulation.

As mentioned above, the arcuate nucleus plays a critical role in feeding regulation and contains the well-studied AGRP and POMC neurons. However, the TN of hypothalamus is an understudied brain region. Recently, Luo et al*.* [12] revealed an important role of the TN in energy homeostasis and revealed a previously unknown circuit mechanism of feeding regulation that operates through orexigenic TN SST neurons. They found that TN in mouse is marked by a dense cluster of SST-positive neurons, which constitutes a hypothalamic neuronal subtype that is distinct from the neurons currently known to support feeding and metabolic regulation. Ghrelin injection induces the activation of SST neurons in TN, which promotes feeding in mice [12].

## Ghrelin influences the hedonic and incentive responses to control appetitive behavior

Ghrelin is secreted by the gut and causes the motivation to consume food. The preprandial rise and postprandial fall in plasma ghrelin levels in humans suggest that it is a hunger signal that promotes meal initiation [80]. Feeding behavior is often separated into homeostatic and hedonic components. Hedonic feeding can be activated by visual or olfactory food cues, which involve brain regions that play a role in reward and motivation, whereas homeostatic feeding is assumed to be under the control of circulating hormones acting primarily on the hypothalamus.

In animals, the behavioral response to such stimuli is mediated by specific neurons in the OFC, amygdala, and striatum [81], which form a part of the mesolimbic reward system that is implicated in motivated behaviors [82]. It has been suggested that although the hypothalamus primarily regulates the homeostatic drive to eat, these other neural circuits integrate environmental and emotional factors to control the ‘hedonic’ drive. Ralevski et al. [83] revealed that intravenous alcohol infusion significantly suppresses ghrelin level in healthy social drinkers, suggesting that ghrelin may have a role in the rewarding mechanism for alcohol. Nonetheless, homeostatic signals access reward-related brain areas to influence the behavior. Ghrelin may modulate the incentive and hedonic aspects of ingestive behavior.

Recently, Dagher and co-workers [16] showed that ghrelin administered intravenously to healthy volunteers increases the neural response to food pictures; this effect is associated with self-rated hunger ratings. The authors demonstrated that ghrelin may augment hedonic and incentive responses to influence food intake. Individuals with higher ghrelin levels are more sensitive to reward and impulsive behavior [84]. However, chronic high-intensity intermittent training has no significant effect on appetite or food reward and plasma AG concentrations in obese individuals [85]. The neuronal substrates, including dopamine, opioids, NPY, orexins, nicotine acetylcholine receptor, glutamate, and endocannabinoids, may also mediate the action of ghrelin on hedonic food and food reward behavior [86].

Ghrelin also increases the response to food pictures by brain areas involved in visual processing, attention, and memory. The pulvinar and fusiform gyri are specifically involved in focused visual attention [87]; the authors also found that ghrelin affects the hippocampus, a structure that, along with the amygdala, is well-known to be involved in memory formation [88]. Moreover, in animals, ghrelin regulates hippocampal spine synapse density and long-term potentiation and enhances spatial learning and memory [89]. However, Kunath et al. [90] did not find any evidence for the potential of ghrelin acting as a short-term cognitive enhancer in humans. It has been found that viewing food pictures does not affect total calorie intake and ghrelin levels, but improves postprandial glucose levels [91].

Brain regions implicated in all of these functions are modulated by ghrelin. The mechanism by which ghrelin acts on the brain is not completely known, but several potential mechanisms have been identified. First, peripheral ghrelin may act on ghrelin receptors in the gut, which then relay information to the brain via the vagus nerve [92], although this pathway is not necessary as total vagal differentiation does not abolish the orexigenic effects of peripherally administered ghrelin. This suggests that circulating ghrelin also acts directly on the brain. A likely region mediating this effect is the hypothalamus, where ghrelin increases the firing rate of NPY/AGRP neurons in the arcuate nucleus [7]. Ghrelin, therefore, appears to modulate the response to food cues via a neural network involved in the regulation of feeding and, most importantly, in the appetitive response to food cues.

## Ghrelin and lipid metabolism

Ghrelin can regulate GH release, food intake, adiposity, and energy metabolism. It promotes adiposity by the activation of hypothalamic orexigenic neurons and stimulates the expression of several fat storage-related proteins in adipocytes, thereby stimulating lipid accumulation [93]. In obese children, AG levels and AG:obestatin ratio are significantly lower, and AG is negatively correlated with triglyceride (TG), low-density lipoprotein-C, insulin, and homeostatic model assessment of insulin resistance [94]. Ghrelin is an important factor linking the CNS with peripheral tissues that regulate energy homeostasis and lipid metabolism. Central ghrelin may store energy as fat by altering adipocyte enzyme expression [64]. In rainbow trout, ICV ghrelin treatment modulates lipid metabolism in the liver, resulting in increased lipogenesis and decreased fatty acid oxidation [95]. ICV ghrelin infusion exhibits heavier epididymal WAT, reduced liver glycogen content and TG, and regulation of the genes involved in hepatic lipid and glucose metabolism in mice [96]. Intravenous infusion of ghrelin increases TG, cholesterol, and free fatty acid (FFA) levels in rats, and this process is age-dependent, indicating the central regulation of ghrelin in lipid metabolism [97].

Previous data have also indicated that ghrelin exerts direct peripheral effects on adipocyte and lipid metabolism. In humans, ghrelin infusion significantly increases TG levels in patients with anorexia nervosa, resulting in increased hunger sensation and daily energy intake [98]. This result provides a potential therapeutic ability of ghrelin in patients with anorexia nervosa. Ghrelin increases WAT mass in selective abdominal depots by a GHSR1-dependent mechanism [99]. GHSR ablation reduces lipid uptake and lipogenesis in WAT [100]. Taken together, these data show that GHSR is an important regulator of lipid metabolism and energy expenditure. Cai et al*.* [101] indicated that ghrelin plays a modulatory role in taste sensitivity and lipid metabolism. Furthermore, ghrelin exerts protective effects on fructose corn syrup-induced adiposity and insulin resistance [102]. Moreover, ghrelin signaling has an important role in macrophage polarization and adipose tissue inflammation during aging [103]. The effects of ghrelin in apoptosis of human visceral adipose tissue have also been reported, and it has been found that both AG and desacyl ghrelin reduce TNF-α-induced apoptosis and autophagy in human visceral adipocytes [104]. Ghrelin also suppresses endothelial apoptosis under high glucose/high lipid conditions via inhibiting JNK1/2 and p38 signaling in humans [105], suggesting an anti-inflammatory role of ghrelin in humans. A previous study has reported that the mammalian target of rapamycin (mTOR) signaling pathway activates peroxisome proliferator-activated receptor γ (PPARγ) to induce adipogenesis [106]. Ghrelin affects hepatic lipid metabolism and stimulates lipogenesis in the liver by direct activation of its receptor on hepatocytes, and the mechanism is also involved in the mTOR–PPARγ signaling pathway [107]. Mao et al. [108] observed the therapeutic effect of ghrelin on nonalcoholic fatty liver disease (NAFLD) and found that subcutaneous ghrelin administration reduces TG content in the HFD group *in vivo* and in the FFA group *in vitro*. The mechanism involves ghrelin-upregulated autophagy via AMPK/mTOR restoration and inhibits translocation of NF-κB into the nucleus [108]. In a cross-sectional study on patients with T2DM, serum AG levels increased in T2DM patients with NAFLD compared with those in patients without NAFLD, demonstrating that elevated AG levels are associated with NAFLD, and an increase in AG over 0.52 ng/ml could be used as a diagnostic marker for NAFLD detection in patients with T2D [109]. Ezquerro et al. [114] reported that DIO rats develop hepatosteatosis and exhibit decreased circulating desacyl ghrelin without changes in AG. SG induces a dramatic decrease in desacyl ghrelin level but increases the AG/desacyl ghrelin ratio. Moreover, both AG and desacyl ghrelin significantly increase TG content in primary rat hepatocytes. In rain trout, ghrelin increases the mRNA levels of lipoprotein lipase, fatty acid synthase, and PPARβ, thus, stimulating the synthesis of triglycerides and their mobilization [110]. This result indicated that ghrelin seems to be an enhancer of lipid turn-over in the adipose tissue of rainbow trout, and this regulation may at least partly be mediated through autocrine/paracrine signaling. These observations all emphasize the potential function of the ghrelin system in lipid metabolism.

Rafee et al. [111] demonstrated the effect of apolipoprotein B insertion/deletion polymorphism and diet on serum lipids, leptin, and ghrelin levels in patients with T2DM and found that high dietary intake of carbohydrate by *Del-allele* carriers may have a protective role against hyperleptinemia and hyperghrelinemia by increasing the sensitivity of leptin and ghrelin receptors. Intravenous lipid infusion with plasma FFA elevates AG level and AG/total hormone ratio [112]. Ullrich et al. [113] investigated the effects of intraduodenal lipid and protein on plasma ghrelin, PYY, and leptin concentrations in healthy humans and found that both lipid and protein potently suppress plasma ghrelin level compared with the control, and lipid exhibits a much more potent effect to stimulate PYY release but do not stimulate leptin. These results demonstrated that direct infusion of both protein and lipid into the duodenum at isocaloric amounts reflect the normal rate of gastric emptying and potently and comparably suppress ghrelin level.

## Conclusion

Ghrelin and GHSR play important roles in the modulation of energy homeostasis and lipid metabolism, and they widely work in the CNS and multiple peripheral tissues, such as the pancreas and adipose tissue. Therefore, targeting ghrelin and its receptor may provide a therapeutic benefit for people with obesity and glucose–lipid metabolism disorder.

## Abbreviations

AG: acylated ghrelin
AGRP: agouti-related protein
CART: cocaine amphetamine-related transcript
CNS: central nervous system
DIO: diet-induced obesity
DRD1: dopamine receptor-1
FFA: free fatty acid
GH: growth hormone
GHRH: GH-releasing hormone
GHSR: growth hormone secretagogue receptor
GLP-1: glucagon-like peptide-1
GOAT: O-acyltransferase
GSIS: glucose-stimulated insulin secretion
HFD: high-fat diet
ICV: intracerebroventricular
JNK: c-Jun N-terminal kinase
mTOR: mammalian target of rapamycin
NAFLD: nonalcoholic fatty liver disease
NF-κB: nuclear factor kappa B
NPY: neuropeptide Y
OFC: orbitofrontal cortex
POMC: proopiomelanocortin
PPARγ: peroxisome proliferator-activated receptor γ
RQ: respiratory quotient
SCFA: short-chain fatty acid
SST: somatostatin
T2DM: Type 2 diabetes mellitus
TNF-α: tumor necrosis factor alpha
WAT: white adipose tissue
